# The lived experience of breast cancer patients on adjuvant endocrine therapy: Side effects and coping strategies during the first year of medication initiation

**DOI:** 10.21203/rs.3.rs-2497342/v1

**Published:** 2023-01-30

**Authors:** Sameh Gomaa, AnaMaria Lopez, Rachel Slamon, Rita Smith, Elma Telushi, Emmanuel Lapitan, Ginah Nightingale, Suzanne Miller, Kuang-Yi Wen

**Affiliations:** Thomas Jefferson University Hospital; Thomas Jefferson University Hospital; Thomas Jefferson University Hospital; Thomas Jefferson University Hospital; Thomas Jefferson University Hospital; Fox Chase Cancer Center; Fox Chase Cancer Center; Fox Chase Cancer Center; Thomas Jefferson University Hospital

## Abstract

**Purpose:**

Breast cancer in women is the most commonly diagnosed cancer. Adjuvant endocrine therapy (AET) showed consistent improvements in recurrence and survival rates. Adherence to adjuvant endocrine therapy remains essential for improving overall survival in women with hormone receptor (HR) positive breast cancer. However, early discontinuation of medicine is reported to range from 20% to 50%. Poor adherence has been attributed to multiple factors including presence of adverse events. We aim to report the lived experience of breast cancer survivors specifically as regards to side effects, the most reported reason for lack of adherence.

**Methods:**

35 breast cancer survivors on AET were interviewed. Qualitative iterative analysis was conducted using the grounded theory approach with the goal of identifying themes that emerge from the interviews and refining the question probes as needed. A codebook was developed and supplemented with interpretive codes generated through ongoing analysis of transcripts. All transcripts were coded using NVIVO qualitative data analysis software for data interpretations.

**Results:**

Reported side effects associated with AET medications include hot flashes, sexual side-effects, joint pain, stiffness, cognitive function, mood changes, bone mass density decrease and fertility concerns. Women who were on AET more than 6 months reported more side effect concerns. A variety of coping strategies using over the counter medications or alternative medicines and approaches were also discussed.

**Conclusion:**

Tailored and timely information on potential AET-induced side effects and strategies to manage them is needed. In particular, some side effects are more prevalent by medication (e.g., joint pain in those who were taking an aromatases inhibitor). Provision of information to prepare women for the potential side effects of type of AET they are prescribed for would be helpful.

**Implications for Cancer Survivors:**

As AET has been suggested for 10 years to improve surveillance and reduce recurrence, our results have implications for cancer survivors, especially the onsets of side effects and potential ways to manage them as they arise.

## Introduction

Breast cancer in women is the most commonly diagnosed cancer surpassing lung cancer. In 2020. An estimated 2,261,419 new cases were diagnosed in women across the world. In the year 2022 it is estimated that 339,250 women in the United States will be diagnosed with breast cancer. The disease accounts for 1 in 3 of new female cancers annually [1], and 70 to 80 percent of all breast cancer diagnosed are hormone receptor (HR) positive. Women with ER positive or PR positive invasive breast cancer are considered for adjuvant endocrine therapy (AET) regardless of age, lymph node status [2]. The use of AET is supported by cancer clinical trial data with improved rates of recurrence and survival [3], making AET essential in the survivorship period. However, early discontinuation of medicine reported to range from 20% to 50%, conformity to prescribed dose reported to range from 74% to 84% and may diminish over time [4]. Breast cancer patients with hormone positive cancer can have poor adherence regardless of age or stage. Poor adherence has been attributed to multiple factors including presence of adverse events, extremes of age, follow-up performed by general practitioners rather than oncologists, personal health preferences, financial reasons and costs, lack of patient understanding of benefits associated with regular use of medicine [5].

A self-reported study among 538 women showed that adverse effects were prevalent and were the most common reason of AI discontinuation[6]. Adverse effects associated with AET are mainly due to systemic estrogen deprivation[7] resulting in hot flashes, night sweats, poor sleep, and dyspareunia. Musculoskeletal symptoms are common like arthralgia. Estrogen deficiency can lead to osteoporosis, bone fractures, and/or osteopenia. Plus a range of other symptoms, resulting in fatigue, depression, headache, hot flashes and night sweats [8]. Limited qualitative data have been collected in the first year of AET when behaviors to support long-term adherence may be established. Using grounded theory, the authors report the lived experience of breast cancer survivors during the first year of AET use [9]. The aim is an informed outlook on the barriers and facilitators to adherence specifically as regards to side effects, the most reported reason for lack of adherence[5]. The interview data will inform the development of tailored interventions to improve AET adherence during this critical transition phase.

## Methods

### Sample

Sample participants were recruited from the Sidney Kimmel Cancer Center (SKCC) at Thomas Jefferson University and Fox Chase Cancer Center (FCCC) in Philadelphia, Pennsylvania, USA. Participants were women who were 18 years old and older, English speaking, had been diagnosed with primary breast cancer (stage I-III), ER+ or PR +, and were within 1 year of initiating AET. Because the type of AET influence adherence and symptom experienced 0], [11]. We purposely sampled participants who were on an AI or Tamoxifen and compared results of medication duration between patients. The two groups are 6 months or more or less than 6 months, as the distribution mean of patients was around the 6 months mark.

### Interview procedures

IRB approval was obtained for this study from the Thomas Jefferson University IRB committee. Consent was obtained from all patients to participate in the study. A preliminary interview guide, developed from our previous study [12], was used to probe participants on their understanding and expectations of AET, their experience with taking AET as prescribed, their experiences with AET related side effects, and their suggestions on how to improve AET symptom management. Participants were interviewed from December of 2019 into December 2020, interviews were either conducted in-person or remotely over Zoom, to accommodate the constraints of the COVID-19 pandemic.

### Analysis

We are seeking to understand the complexities and nuances of patient’s beliefs and lived experiences hence the qualitative method approach which allow a richer perspective that quantitative methods are limited in exploring. We used a grounded theory approach, which is particularly useful for understanding complex symptom phenomena and their underlying dynamics. The process enabled us to investigate the conceptual symptom themes and facilitate our understanding of the coping process as a whole [13]. Recordings of interviews was transcribed. Iterative analyses was conducted with the goal of identifying themes that emerge from the interviews and refining the question probes as needed. A codebook was developed and supplemented with interpretive codes generated through ongoing analysis of transcripts. All transcripts were coded using NVIVO qualitative data analysis software. Ten interview transcripts were reviewed by four coders to establish the coding schema, the 25 interviews remaining were divided between 2 coders each consisting of 2 reviewers to complete the coding. The team members met on a weekly basis to discuss the codes, emerging themes, coding differences found after each round a coding, discussed a comparison query in NVIVO. After each weekly meeting an updated NVIVO file with updated themes/codes was distributed for subsequent coding. The Kappa values ranged from 0.84 to 1.00 for the codes.

## Results

We have attempted to contact 94 breast cancer patients on AET using referrals from medical oncology by the clinical research coordinator on the study, 40 subjects were recruited for the interviews, and 35 interviews were completed and analyzed. Patients who refused to participate for multiple reasons as being overwhelmed by their cancer experience, showed no interest in participation, had multiple cancers or scheduling conflicts.

Table 1 summarizes participant’s demographic and medical characteristics (N=35). The mean age of participants was 52.5 years (SD= 11.73). The majority of the participants where college graduates (74.2%), were White (68.5%), married (57.1 %). 24 women (68.5%) were diagnosed with stage I breast cancer, 6 with stage II (17.14%) and 3 with stage III (8.5%) and the majority were on aromatase inhibitors (65.5%) versus 12 on tamoxifen (34.2 %). On enrollment, 3 women (8.7%) had been taking their medication for less than 3 months, 13 (37.1 %) were taking the medication for 3–6 months and 19 (54.2%) were taking the medication for 6–12 months.

### Side effects of medication and overtime

Table 2 reported a quantified number of patients experiencing concerning side effects and grouped based on the type and length of medication. In general, patients experienced more side effects the longer the duration of treatment. However, Tamoxifen was associated with earlier sexual side-effects and hot flashes during the first 6 months instead of later down the treatment timeline. The joint pain, stiffness, mood changes, and bone loss were reported after 6 months on the treatment timeline. For women on aromatase inhibitors, sexual issues, hot flashes, joint pain, and stiffness were reported more after the 6 months mark. Mood changes, fertility concerns, and bony aches or concerns were generally equivocal between patients in both duration groups.

Coping strategies have been varied, as elaborated in the results section. Patients cited coping strategies for joint pain the most, followed by hot flashes, supporting that they are the most common in breast cancer patients. Sexual health tips were cited less commonly, and lastly, mood and cognitive tips were mentioned the least.

### Joint pain

Patients described their personal experience with the pain and how pain persists daily, impacting their daily life. A patient likened it to exercise pains.

“It’s just everything hurts all the time. I feel like I just exercised yesterday, but it’s every day that I am exercising, so we don’t even have the benefit of it.”

(Tamoxifen, more than 6 months)

Patients shared their struggles and confusion about whether to attribute the pain to the medication or their comorbidities.

“….the joint pain is really my only problem. I have arthritis anyway….”

(Letrozole, more than 6 months)

“…..So how do I know that it was the result of the medication I was on, that’s one of the reasons why I had to stop taking Letrozole, so we could narrow down the actual cause of my joint pain and the muscle pain “

(Letrozole, less than 6 months)

However, the pain could be so severe that some patients had to pause the medication based on their care team’s recommendation

“… my side effects of joint pains are really severe. So, they asked me to stop the medication for about three weeks.”

(Letrozole, more than 6 months)

### Stiffness

Stiffness represented a challenge to most patients in its often nuanced, a patient described the feeling of stiffness as if prematurely aging.

“…I think the stiffness I get, I feel very tight. My body. So when I go to get up after sitting for a little while, I feel like an old lady….”

(Anastrozole, less than 6 months)

It tends to have a progression throughout the day increasing early in the day and, a resemblance to other comorbidities

“…. it’s like arthritis, as I’m starting my day, it goes away I feel like I have arthritis when I wake up in the morning and then my fingers they’re cramping and achy and sometimes I drop things that I don’t normally drop….”

(Anastrozole, less than 6 months)

### Self-reported strategies for coping with pain and stiffness

Patients employed multiple strategies to cope with their pain and stiffness. Analgesics were often the first approach.

“… I would take Tylenol and that seemed to work..…”

(Letrozole, less than 6 months)

others opted for non-steroidal anti-inflammatory medications

“…. As far as the joint pain goes, I do take Advil or Naproxen. I take three a day.…”.

Anastrozole, more than 6 months)

Some also reported that varying the medication intake time helped with the pain; by taking the medication at night, they avoid the pain by day.

“…. so I take it at night, so it occurs during the night, and I don’t have it so much during the day.…”

(Letrozole, less than 6 months)

Massage was reported as an effective solution in dealing with joint pain.

“..… I was like, wow, I just get like a massage. I’ll just massage myself and um, drink plenty of water….”

(Exemestane, less than 6 months)

Stretching and physical activities were helpful routines to reduce pain and stiffness.

“..… Yes. I try to do yoga. Oh, um and workout. Um, I only do that once or twice a week on a studio, and then I try to work out two or three times a week. Walking helps tremendously. Yeah. I try to walk my dogs twice a day.…”

(Anastrozole, less than 6 months)

Seeking help by the cancer care team, participants shared experiences with non-physician health professionals like physical therapy.

“…I’m not managing them very well. The pain I’m managing by seeing physical therapy. So that’s why you see the hand brace. They haven’t dealt with this hand yet because I can only deal with one hand at a time.…”.…

(Anastrozole, less than 6 months)

### Hot flashes

Women reported the quality and quantity of their hot flashes, especially how disruptive they are during sleep and impact on daily activities.

“.… I just get overheated, like really fast I’ll just start sweating and it’s bad at nighttime, so I’m, constantly tossing and turning at night, so that bothers me because I have to get up in the morning to work. ..…”

(Anastrozole, less than 6 months)

“…. Almost everything that I do, it doesn’t seem like I have the tolerance to actually do it more than 15 minutes because these hot flashes are coming every 10, 15 minutes or so and you get annoyed.…”

(Tamoxifen, less than 6 months)

Surprisingly one patient described the hot flashes as chills

“.…I’m very very cold all the time at home. I have to put my heat on 75 because I’m cold, which is a bad side effect. And I don’t know if it came from that particular medicine or not but I didn’t have the chills like I do now until I started taking it.…”

(Tamoxifen, less than 6 months)

### Self-reported strategies coping with hot flashes

The most common technique reported to reduce night sweats is to take the AET earlier in the day

“Taking it in the morning, I felt like, and even if I take it too early in the night, if I take it dinnertime, I feel like I get night sweats really bad”

(Anastrozole, less than 6 months)

Selective serotonin reuptake inhibitors have helped.

“…. They did give me Lexapro. I don’t know if you know that they gave me Lexapro five milligrams, which is used off label…..”…..

Several women commented on the positive effects they had experienced from receiving acupuncture.

“…. Um, my main way to work with these is, um, acupuncture. J do acupuncture regularly………”

(Letrozole, less than 6 months)

### Sexual health is a non-discussed symptom

Many patients pointed out that sexuality was a very private matter and felt uncomfortable sharing that even with their significant others or their doctors. Some did not receive information from healthcare providers regarding sexual issues and complications caused by AET-induced menopause symptoms.

“… Yeah. Because J think that’s probably a big concern, J mean, it’s uncomfortable to talk to your doctor about that stuff. A lot of times J think women just feel like myself I’ll just figure it out myself, or you know we’ll just work through it. J don’t know if this is really addressed the way it should be.”

(Anastrozole, less than 6 months)

“…..J have, a lot of dryness. So J went through menopause about three years ago, but J really feel that since J’ve been on this pill J was diagnosed with atrophic vaginitis “

(Anastrozole, more than 6 months)

### Self-reported strategies for coping with physical, sexual issues

Suppositories or personal lubricants were the most effective in improving vaginal dryness during intercourse.

“…. I use K-Y jelly, for sexual intercourse, you know, for relations with my husband. Um, and that works fine for me.…”

(Anastrozole, more than 6 months)

### Mood changes

Patients expressed mood swings on AET medications, arousing concerns by their spouses.

“… My Husband was asking questions too, because of my mood swing. J have a lot of, you know, mood swings, ….”

(Anastrozole, more than 6 months)

Breast cancer survivors deal with a lot of anxiety in general or about minute details in their lives

“..… I was having major anxiety attacks, the doctor finally put me on some Prozac ……”.

(Tamoxifen, more than 6 months)

“…Sometimes I can feel myself getting agitated, like I can feel myself getting worked up about something that I don’t need to get worked up about..…”.

(Tamoxifen, more than 6 months)

A patient described her mood swings through the day and how changeable across the spectrum of emotions they can be.

“ ……Sometimes I can feel myself getting agitated, like I can feel myself getting worked up about something that I don’t need to get worked up about..…”

(Anastrozole, more than 6 months)

A patient shared how she was impacted emotionally and mentally

“ …And then I started getting the anger. And then I felt like I could not relax. I could not concentrate.…”

(Anastrozole, more than 6 months)

### Self-reported strategies coping with mood changes

For mood swings, one patient had her physician eventually prescribe SSRIs

“..you need to sit down with yourself and take a break, the doctor finally put me on some Prozac..”

(Anastrozole, more than 6 months)

Deep breathing and relaxation exercises help alleviate stress and anguish.

“… deep breathing, just nice deep breath to slow my breathing and just center myself.…”

(Tamoxifen, less than 6 months)

Routine exercise is a central coping mechanism for many patients dealing with negative moods and stress.

“I also exercise a lot. I exercise every single day. I do stretching, I do some yoga. I might do spin classes on a bike. I might do strength training. If it’s nice day outside, I might go for a run. I might ride a mile or two. So that helped me tremendously.”

(Tamoxifen, less than 6 months)

Some how a patient found resolve in community involvement and being in service to others

“……. I indulged in that I become a support system to other people. I also support these two little girls who’s going through cancer and they are the ones that really get me off my feet…”

(Anastrozole, more than 6 months)

## Discussion

While this study was not intended to statistically compare the subgroups (Tamoxifen vs. AI or medication duration < 6-month vs.> 6-month), we observed that participants’ perspectives across subgroups bring novel insights. In general, side effects vary based on the type and duration of the medication. This research aims to understand The lived experience of breast cancer survivors on AET. To have an informed outlook in a more intimate one-on-one interview style to find what motivates the patient to maintain adherence and the barriers to adherence, specifically regarding side effects of the medication, the most reported reason for lack of adherence[5]. The patients shared their concerns and ways to cope with them. We found a common theme; despite the side effects, patients continue to adhere to the medication as the benefits of survival and recurrence prevention outweigh the side effects. Patients have learned to cope with the side effects either with the support of their care team or through personal lifestyle and routine changes that are most fitting to their lifestyles.

The most pressing side-effect was joint pain; a study reported arthralgia [13] in 30.2% of patients. Women expressed that they were hindered by pain and stiffness of different severities; some perceived pain to be minor with minimal interference in their daily activities, and others were disabled. Consistent with the literature, the medication aggravates preexisting joint pain or arthritis[14]. Hot flashes are a commonly reported symptom and occur in up to 70% of patients on AET[15]. It interfered with the patient’s sleep in the form of excessive sweating or the feeling of being overheated. Patients discussed the effects of AET on their sexuality as vaginal dryness and lack of desire were potentially induced by AET, as supported by the literature[16]. Vaginal dryness risk is associated with higher tamoxifen levels [15]. Women also expressed their frustration in not being able to and not being comfortable in sharing their experience with their spouses and their care team and felt alienated. On Mood Changes & Impact on cognition Patients reported having mood swings, anxiety or isolation, and dealing with mental issues. Tamoxifen has been implicated in slower cognitive processing than AI’s[15].

**Table:** 

Mitigation strategy	Complaint
OTC pain medications	Joint pain and stiffness
Massages with different topical creams	
Exercise, stretching, yoga, bike riding, and walks	
Physical therapy	
Medication Pause or time variation	
Acupuncture	Hot flashes
Medication Pause or time variation	
Personal lubricants and suppositories	Vaginal Dryness
Breathing exercises	Mood Changes
Charity and community involvement	

The patients used the following strategies to deal with the side effects. Pain medications such as NSAIDs and Paracetamol were commonly reported to use for relieving joint pain and stiffness. However, literature proved their inefficacy compared to serotonin and norepinephrine reuptake inhibitors [15]. Massages with different topical creams have been successful in relieving pain and stiffness for our patients; however, there is minimal proof of effectiveness[17][18], and it is generally less effective than mind-body therapies like yoga [19]. Exercise, stretching, yoga, bike riding, and walks have all been reported helpful in our sample in relieving joint pain and stiffness, hot flashes, and alleviating mood change and anxiety, consistent with the literature[20]. Physical therapy has been reported effective in reducing joint pain and stiffness. According to the American society of clinical oncology, there have been limited recommendations to start physical therapy before initiating treatment to address limitations in movement, strength, and balance[21]. Acupuncture was perceived as positive in controlling hot flashes [15], [22]. Personal lubricants and suppositories were reported to be helpful for vaginal dryness [23], consistent with the American College of Obstetricians and Gynecologists’ recommendations [13]. Breathing exercise: was a tool some used with success to relieve mood changes. As per NCCN, it is used to relieve hot flashes with minimal evidence[5]. Medication change or discontinuation: was a last resort measure if the symptoms were intolerable and debilitating. Other personal scoping strategies included during the day the patients dressed in layers changed as needed, used fans and cold drinks to counteract the hot flashes. Medication time variation. Some patients took the medication at the morning time to avoid the night hot flashes. Others took the medication at night time to avoid the joint pain during the day. Charity and community involvement cited by one patient, she was keeping active in her community with charity work and has helped her deal with mood changes and improved overall wellbeing. A continuous tailored support system for each patient will be needed to connect the patient with all resources of their cancer care team, which entails initiating sensitive discussions like sexuality with patients and setting expectations after initiating the treatment. Most patients will avoid disclosing complaints to their care team or their partners about personal concerns resulting in alienation. Ongoing supportive programs such as exercise, yoga, and other alternative medicine approaches should be considered to promote patient-centered survivorship care. Developing strategies to track side effects and their severity would be helpful to allow better doctor-patient communication and decision-making about medication choices.

### Study strengths and limitations

The current study is one of the few qualitative studies on the experience of breast cancer patients on AET during the first year of survivorship. We used qualitative methodologies to provide more detailed information about their experience with side effects and management. Including a sample of patients taking either an AI or Tamoxifen and who were at a varying medication timeline during the first year of survivorship is a strength. Although our sample was diverse, the small sample size and the purposive sampling strategy mean that the results might not be generalizable to a broader population.

## Conclusion

This qualitative study had shed light on the side effects the patient experience, which can negatively impact adherence to AET, and modifiable intervention factors that can promote adherence and side effect management. These include the need for a continuous tailored support system that is not reactive but proactive.

## Figures and Tables

**Table 1.** Demographic data

Variable		N=35	Percentage
Age, years: M (SD)		52.5 (11.73)	
Race	White	24	68.5 %
	Black or African American	10	28.5 %
	Asian	1	2.8 %
Ethnicity	Non-Hispanic	34	97.1 %
	Hispanic	0	0 %
	Unknown	1	2.9 %
Marital status	Married	20	57.1 %
	Single / Divorced	15	42.9%
	Widowed	0	0 %
Education level	High School	9	25.7 %
	College Graduate	24	74.2 %
	Post Graduate	2	5.7 %
Breast cancer stage	I	21	68.5 %
	II	6	17.14 %
	III	4	8.5 %
Medication	Letrozole	5	14.2 %
	Anastrozole	15	42.8 %
	Exemestane	3	8.5 %
	Tamoxifen	12	34.2 %
AI medication duration:	Less than 3 months	3	8.7 %
	3–6 months	13	37.1 %
	6–12 months	19	54.2 %

**Table 2:** side effects and the number of patients experiencing the side effects in the transcripts.

	Participants on Tamoxifen (11)		Participants on AI (20)		Total (31)
	Months on med = Less than 6 months 5 (45.5%)	Months on med = 7–12 months 6 (54.5 %)	Months on med = Less than 6 months 7 (35%)	Months on med = 7–12 months 13 (65%)	
SE Concerns related to medication adherence/belief	4 (80%)	5 (83.3%)	5 (25%)	10 (76.9%)	24 (77.4%)
Sexual health	3 (60 %)	2 (33.3%)	1 (14.3%)	3 (23.1%)	9 (29%)
Hot flashes	3 (60 %)	2 (33.3%)	5 (25%)	6 (46.2%)	16 (51.6%)
Joint pain-Stiffness-Aches	2 (40%)	3 (50%)	6 (85.6 %)	9 (69.2%)	20 (64.5%)
Mood changes	1 (20%)	2 (33.3%)	1 (14.3%)	3 (23.1%)	7 (22.6%)
Fertility	1 (20%)	0	1 (14.3%)	1 (7.7%)	3 (9.6%)
Bone mass decrease concerns	0	0	1 (14.3%)	1 (7.7%)	2 (6.5%)
**Reported SE management strategies**	2 (40%)	3 (50%)	6 (85.6 %)	7 (53.8%)	18 (58.1%)
Sexual health Vaginal Suppositories, Personal lubricant.	2 (40%)	1 (16.6 %)	0	2 (15.4%)	5 (16.12%)
Hot flashes SSRI Varying the time of medication Acupuncture	3 (60 %)	3 (50%)	3 (42.9%)	4 (30.1%)	13 (41.93%)
Joint pain-Stiffness-Aches Analgesics Varying the time of medication Exercise Massage Physical Therapy	0	3 (50%)	4 (57.1%)	7 (53.8%)	14 (45.2%)
Mood changes Exercise Deep breathing excercises	1	1 (16.6 %)	0	1 (7.7%)	3 (9.6%)
